# Siderophore-Mediated Iron Dissolution from Nontronites Is Controlled by Mineral Cristallochemistry

**DOI:** 10.3389/fmicb.2016.00423

**Published:** 2016-03-31

**Authors:** Damien Parrello, Asfaw Zegeye, Christian Mustin, Patrick Billard

**Affiliations:** ^1^Laboratoire Interdisciplinaire des Environnements Continentaux, UMR 7360 Centre National de la Recherche Scientifique - Université de LorraineVandœuvre-lès-Nancy, France; ^2^Civil and Environmental Engineering, University of MissouriColumbia, MO, USA

**Keywords:** siderophore, pyoverdine, *Pseudomonas aeruginosa*, bioweathering, iron mobilization, nontronite, clay

## Abstract

Bacteria living in oxic environments experience iron deficiency due to limited solubility and slow dissolution kinetics of iron-bearing minerals. To cope with iron deprivation, aerobic bacteria have evolved various strategies, including release of siderophores or other organic acids that scavenge external Fe(III) and deliver it to the cells. This research investigated the role of siderophores produced by *Pseudomonas aeruginosa* in the acquisition of Fe(III) from two iron-bearing colloidal nontronites (NAu-1 and NAu-2), comparing differences in bioavailability related with site occupancy and distribution of Fe(III) in the two lattices. To avoid both the direct contact of the mineral colloids with the bacterial cells and the uncontrolled particle aggregation, nontronite suspensions were homogenously dispersed in a porous silica gel before the dissolution experiments. A multiparametric approach coupling UV-vis spectroscopy and spectral decomposition algorithm was implemented to monitor simultaneously the solubilisation of Fe and the production of pyoverdine in microplate-based batch experiments. Both nontronites released Fe in a particle concentration-dependent manner when incubated with the wild-type *P. aeruginosa* strain, however iron released from NAu-2 was substantially greater than from NAu-1. The profile of organic acids produced in both cases was similar and may not account for the difference in the iron dissolution efficiency. In contrast, a pyoverdine-deficient mutant was unable to mobilize Fe(III) from either nontronite, whereas iron dissolution occurred in abiotic experiments conducted with purified pyoverdine. Overall, our data provide evidence that *P. aeruginosa* indirectly mobilize Fe from nontronites primarily through the production of pyoverdine. The structural Fe present on the edges of NAu-2 rather than NAu-1 particles appears to be more bio-accessible, indicating that the distribution of Fe, in the tetrahedron and/or in the octahedron sites, governs the solubilisation process. Furthermore, we also revealed that *P. aeruginosa* could acquire iron when in direct contact with mineral particles in a siderophore-independent manner.

## Introduction

Iron is an essential nutrient, playing a central role in the growth and the development of most living organisms. It is required for several fundamental enzymatic processes including respiration, photosynthesis and DNA synthesis. Most microorganisms require Fe concentration around 10^−7^ to 10^−5^ M to support optimal growth (Andrews et al., 2003). Although widespread in the environment, Fe is very insoluble in oxic conditions at circumneutral pH, which tends to limit the concentration of dissolved Fe (Cornell and Schwertmann, 2004). For instance the solubility products of common iron oxides such as goethite, hematite and ferrihydrite are extremely low ((Fe^3+^).(OH)^3^ = 10^−39^–10^−44^ M), thus the bioavailable Fe concentration falls much below the metabolic needs of bacteria. To overcome the low abundance of free Fe in aerobic environments, bacteria have developed different strategies such as the excretion of protons and/or low molecular weight organic acids (e.g., citrate, oxalate) (Banwart et al., 1989). These metabolites contribute to mineral weathering by decreasing the pH or by forming complexes with surface ions and subsequently solubilizing the Fe from the mineral lattice by either proton-promoted or ligand-promoted dissolution (Balland et al., 2010).

The synthesis and the excretion of siderophores is another important avenue used by bacteria for Fe acquisition in iron-limited environments (Powell et al., 1980; Saha et al., 2013). Siderophores are low molecular weight organic molecules, with extremely high affinities for Fe(III) up to 10^52^ M^−1^ and as such they are capable to solubilize Fe(III) from minerals lattices. These multi-dentate ligands are considered as one of the main molecules involved in the Fe uptake in the rhizosphere (Powell et al., 1980; Hider and Kong, 2010; Schalk and Guillon, 2013). For instance, *Pseudomonas aeruginosa*, a Gram-negative bacterium commonly found in soil and water, excretes two major siderophores, pyoverdine and pyochelin, under iron deficient conditions (Cox et al., 1981; Brandel et al., 2012; Schalk and Guillon, 2013). Pyoverdine is a dihydroxyquinoline-derived chromophore to which a short peptide chain is attached which binds Fe^3+^ in a 1:1 stoichiometry with a stability constant for Fe(III) around 10^32^ M^−1^ (Albrecht-Gary et al., 1994). On the other hand pyochelin is derivate from thiazoline [2(2-*o*-hydroxy-phenyl-2-thiazolin-4-yl)-3-methylthiazolidine-4-carboxylic acid)] (Cox et al., 1981), which binds Fe^3+^ in a 2:1 stoichiometry with a stability constant for Fe(III) around 10^17^ M^−1^ (Brandel et al., 2012).

The dissolution of Fe(III) bearing minerals such as iron oxides, clay minerals, triggered by siderophores has been the subject of numerous studies (Albrecht-Gary and Crumbliss, 1998; Cheah et al., 2003; Kraemer, 2004; Dehner et al., 2011) and have highlighted different key parameters. For instance, efficiency of Fe(III) uptake by siderophores was enhanced in the presence of organic acids such as oxalate, a common metabolite resulting from bacterial activity. This suggested synergic action consisted, on the one hand, in the labialization of the structural Fe by oxalate and on the other hand, in the detachment of the labile pool of Fe(III)-oxalate surface complexes by siderophores, thus driving the reaction toward more dissolution (Kraemer, 2004). Interestingly, an additive effect rather than a synergic one was observed when ascorbate, a reducing agent, was used instead of oxalate, showing the complex interplay between siderophores and organic molecules during the dissolution of structural Fe(III) (Cheah et al., 2003; Dehner et al., 2010). Additionally, Dehner et al. (2011) have shown that the uptake of Fe(III) by *P. mendocina* from hematite was governed by the particles size. Indeed, Fe(III) associated with particles less than a few tens of nm was more bioavailable than Fe(III) associated with larger particles emphasizing the significance to probe particle size when assessing bioavailability. Together these studies stressed that mineral weathering was a combination of various and complex set of interactions between the mineral and the bacteria. These complex biotic and abiotic interactions likely overcome thermodynamic and kinetic constraints imposed by the mineralogy, the texture and crystallo-chemistry of the mineral, leading to the release of structural Fe, which becomes available for microbial uptake.

Although iron oxides are the ultimate source of Fe, iron bearing clay minerals can also play the role of Fe pool for bacterial metabolism. Recent experiments showed that structural Fe(III) in smectite, a 2:1 phylosilicate, was available for bacterial uptake (Haack et al., 2008; Kuhn et al., 2013; Ferret et al., 2014). However, there still remains a gap in our knowledge whether the structural Fe(III) site occupancy influences the bioavailability of Fe. NAu-1 and NAu-2 are two nontronite, hydrous Fe(III) bearing di-octahedral clay minerals. They are 2:1 phyllosilicates of similar composition with two tetrahedral sheets per octahedral sheet. The structural Fe(III) are located in both the tetrahedrons and in the octahedrons for the two nontronites, the main difference being that 2% of total iron is located in the tetrahedral sheet for NAu-1 against 8% for NAu-2.

The aim of this study was therefore to study the bioaccessibility of structural Fe in these two nontronites, NAu-1 and NAu-2, to determine whether crystallographic sites (tetrahedrons vs. octahedrons) control its uptake by *Pseudomonas aeruginosa* PAO1.

## Materials and methods

### Bacterial strains, plasmids, and culture conditions

The following *P. aeruginosa* PAO1 strains were used in this study: PAO1 wild-type (ATCC 15692), PAO1-Δ*pvdA* (Ochsner et al., 2002) deficient in pyoverdine production and PAO1 Δ*pvdD* Δ*pchEF* (Ghysels et al., 2004) impaired in the production of both pyoverdine and pyochelin. *E. coli* DH10B served as a host for cloning purposes. The plasmid pPROBE-bfrB which contains an iron responsive *bfrB*-*gfp* gene fusion was constructed as described previously (Parrello et al., 2015) except that the *bfrB* promoter region was cloned in pPROBE-NT (Miller et al., 2000). Bacteria were routinely grown aerobically on LB agar or in LB broth at 37°C. Deferrated Casamino Acids medium (DCAA medium) (Visca et al., 1992) was used for mineral weathering experiments. When required, the medium was supplemented with kanamycin at final concentrations of 40 μg.mL^−1^ for *E. coli* or 500 μg.mL^−1^ for *P. aeruginosa*.

### Preparation of nontronite embedded in hybrid silica gels

Two nontronites, NAu-1 and NAu-2, purchased from the Source Clays Minerals repository at Purdue University were used for bioweathering experiments. NAu-1 and NAu-2 contain Fe(III) in different structural sites and are defined by the structural formula (Si_6.98_Al_0.95_ Fe_0.07_)(Al_0.36_Fe_3.61_Mg_0.04_)O_20_(OH)_4_Na_1.05_ and (Si_7.55_Al_0.16_Fe_0.29_) (Al_0.34_Fe_3, 54_ Mg_0.05_)O_20_(OH)_4_Na_0.72_, respectively. The average size of the particles used in the present study was 460 nm in length, 60 nm in width for NAu-1, 368 nm in length, 93 nm in width for NAu-2, and around 1 nm thickness for both nontronites (Michot et al., 2008). Hybrid silica gels were prepared by immobilization of nontronite particles in a microporous, tetraethylorthosilicate- (TEOS, Fluka) derived silica gels by a sol-gel technique as described previously (Grybos et al., 2010, 2011; Oulkadi et al., 2014). For this study, the procedure was automated and silica gels were produced in 96-wells microplates (MTP 96/F-bottom, Eppendorf) with the use of epMotion 5070's automated pipetting system (Eppendorf). The gels had a volume of 0.16 ml and contained 3.3 g L^−1^ of either NAu-1 or NAu-2, or both nontronites at concentrations ranging from 0 to 3.3 g L^−1^ and NAu-1/NAu-2 ratios ranging from 0 to 100%.

### Bioweathering experiments

The nontronite weathering ability of *P. aeruginosa* was determined by incubating bacteria with nontronite embedded in hybrid silica gels in different conditions and by further measurement of iron release and production of organic acids and pyoverdine. *P. aeruginosa* strains were grown overnight in LB broth. The cells were collected by centrifugation, washed twice with DCAA and the cell pellets were suspended in the same medium at an optical density at 600 nm (OD_600_) of 1.

To measure the kinetics of organic acids production, suspensions of PAO1 WT and Δ*pvdA* mutant strains were separately inoculated to an OD_600_ of 0.1 in 4 ml of DCAA medium supplemented with four 0.16 ml silica gel plugs containing NAu-1 or NAu-2 particles at 3.3 g L^−1^ Incubations were performed in triplicate at 37°C with shaking at 260 rpm for 60 h. After 2, 6, 12, 36, and 60 h of incubation, 100 μl of each culture were diluted in 900 μl of ultrapure water and centrifuged at 13,500 × g for 5 min. Supernatants were then filtered with 0.22 μm pore size filters (Millipore) and stored at −20°C before organic acids quantification.

To assess the respective role of nontronite structure and pyoverdine production in the bio-weathering process, suspensions of PAO1 WT and Δ*pvdA* mutant strains were inoculated to OD_600_ of 0.1 in tubes with 2 ml of DCAA and four silica gels plugs containing 25 combined concentrations (i.e., 0, 0.83, 1.65, 2.48, and 3.3 g L^−1^) and ratios (i.e., 0, 25, 50, 75, and 100%) of NAu-1/NAu-2 particles. The suspensions were incubated at 37°C with shaking for 24 h before determination of iron and pyoverdine concentration. This experiment was conducted in duplicate.

### Analytical methods

Organic acids were quantified from weathering solutions using an ion chromatograph (ICS 3000, Dionex corp.) equipped with an Ion Pac® column (AS 11 HC, Dionex corp.) according to a KOH gradient of 0.9–60 mM over 50 min at a flow rate of 1.3 mL per min. Standard solutions (0.1 mM) used were sodium salts of acetate, citrate, formate, gluconate, D,L-malate, oxalate, propionate, succinate, (Sigma Aldrich), lactate, and malonate (Fluka). Measurement uncertainty was lower than 0.5% for all organic acids and the detection limit was close to 0.1 ppm.

Iron concentration in weathering solutions was measured spectrophotometrically at 565 nm by adding 20 μl of Ferrospectral (Merck, Millipore) dye indicator to 200 μl of 0.22 μm filtered culture supernatants. The fraction of iron adsorbed onto silica gels and embedded on mineral surfaces was estimated by treating 1 volume of silica gel with 2 volumes of KCl (1 M). After 1 h of incubation at 28°C, Ferrospectral dye indicator was added as described above. The fraction of iron precipitated onto silica and mineral surfaces was estimated by treating 1 volume of silica gel with 2 volumes of hydroxylamine solution (0.2% NH_2_OH in KCl 1M) for 1 h at 28°C before addition of Ferrospectral.

Concentration of pyoverdine in culture supernatants was quantified spectrophotometrically by measuring the absorbance at 400 nm with ε = 19 000 M^−1^ cm^−1^ (Meyer and Abdallah, 1978).

In addition to the above described mono-wavelength measurement of iron and pyoverdine, the production patterns for these two compounds were determined by means of a recently developed approach that couples spectroscopy with blind spectral decomposition of the spectral data by the Candecomp/Parafac (CP) model (Parrello et al., 2015). Briefly, 200 μl of filtered supernatants from cultures grown with different concentrations and ratios of NAu-1/NAu-2 particles were transferred in 96 well microplates. For pyoverdine profiling, changes in absorption spectra were monitored by scanning the samples from 300 to 900 nm in 2 nm steps and with a 2 nm bandwidth using a FLX-Xenius spectrophotometer (SAFAS, Monaco). For iron dissolution profiling, similar absorbance spectra were performed on both culture supernatants and solutions from KCl- and hydroxylamine-treated silica gel plugs after Ferrospectral addition. Raw absorption data were collected and exported for further signal processing with Candecomp/Parafac algorithms running under Matlab software.

### Blind spectral decomposition by Candecomp/Parafac

The multi-way analysis of spectral data by CP algorithm provided multilinear decomposition of a data matrix without any a priori information about the spectra of the absorbing compounds. It was assumed that absorption spectra from different batches are indicative of various mixtures of R components. Each of the R absorbing component spectrum, termed as the rth source (r = 1, …, R), is mathematically represented by a vector s_r_ = [s_r1_…s_rN_]^T^ made of N entries, i.e., the absorption intensities at each wavelength. The acquisition of absorption spectra as a function of two crossed parameters (i.e., the concentration of nontronite and the NAu-1/NAu-2 ratio *in silica* gels) generated a three-way data array X (three-order tensor; nontronite concentration x NAu-1/NAu-2 ratio x wavelength), which can be expressed by the following tri-linear CP mode:

(1) $$\begin{array}{l} X_{m,p,n}=\overset{R}{\underset{r=1}{\sum }}a_{rm}\;b_{rp\;}s_{rn}+E_{m,p,n} \end{array}$$

where *R* is the number of spectral sources (i.e., decomposition rank) and E is the residual error term (three-order tensor) including experimental error, signal noise or “non-linear” component behavior. An equivalent representation of Equation (1) is given by:

(2) $$\begin{array}{l} X=\left[\left[A,B,S\right]\right]+E \end{array}$$

where the three matrices **A** (*M*-by-*R*), **B** (*P*-by-*R*) and **S** (*N*-by-*R*) are respectively obtained by stacking the vectors **a**_*r*_, **b**_*r*_ and **s**_*r*_. The matrices **A** and **B** in Equation (2) characterize the behavior of the R sources according to the two experimental parameters. The matrix **S** characterizes the spectral shape of the identified sources. The rank *R* of decomposition is assessed experimentally by observing the decrease of the energy of the energy component E. The retained value is obtained after successive trials using possible values of *R* around the value of *R* found before. The energy component represents the level of contribution of each source in the explained variation (the fitting accuracy of the raw three-way dataset). Thus we can soundly define that if the nth supplementary source is 2 log lower the most explicative source, the optimal number of sources is n-1.

The main interest of the CP decomposition comes from its good uniqueness properties, meaning that the matrices **A, B**, and **S** can be uniquely estimated from the data X, i.e., the proposed mathematical model actually describes what happens physically. Another very interesting feature of the CP decomposition is the possibility to extract signals of interest from the background noise, allowing the analysis of spectra obtained from complex environments.

### Microscopy and image analysis

The fluorescence of individual cells of PAO1 Δ*pvdD* Δ*pchEF*/pPROBE-bfrB cultured for 48 h in DCAA medium containing colloidal suspensions of either NAu-1 and NAu-2 (3.3 g L^−1^) was quantified by fluorescence microscopy. Images were acquired on a Nikon Eclipse 80i microscope with Plan Fluor X 40 objective and FITC (ex: 465–495 nm; em: 515–555 nm) filter, and equipped with an Intensilight C-HGFiE fluorescence light source and a digital sight DS-Qi1MC camera. Quantification of GFP fluorescence was performed using the NIS-Elements BR software (version 3.2, Nikon) by encircling bacterial cells and measuring the mean pixel intensity. Image adjustments such as changes of contrast and brightness were applied equally across the entire image.

## Results and discussion

### Bio-dissolution of structural iron of colloidal size NAu-1 and NAu-2

Iron bio-dissolution from colloidal NAu-1 and NAu-2 entrapped in a porous silica gel was investigated using a multi-parametric approach where the NAu-1 and NAu-2 ratio was varied across a range of particle concentrations. Previous experiments indicated that the mineral trapping procedure neither affected the mineral structure nor prompted its dissolution as no iron was detected in the media in the absence of bacteria (Grybos et al., 2010, 2011). Additionally, the nontronite particles included in the silica gel were homogeneously dispersed and in a colloidal form, thus the reactivity of the mineral was not likely affected by a change of its surface area (due to aggregation), or to physical defects (stacking faults, mixed layering). Another critical feature was the absence of physical contact between the mineral and the bacteria cells once the mineral was trapped in the silica gel (Grybos et al., 2010, 2011). Therefore, this experimental approach allowed assessing the dissolution of Fe(III) from colloidal NAu-1 and NAu-2 without a direct contact with *P. aeruginosa* cells. The dissolution, if any, would be more likely related to the diffusion of organic molecules through the micro-porous silica gel.

**Figure 1:**
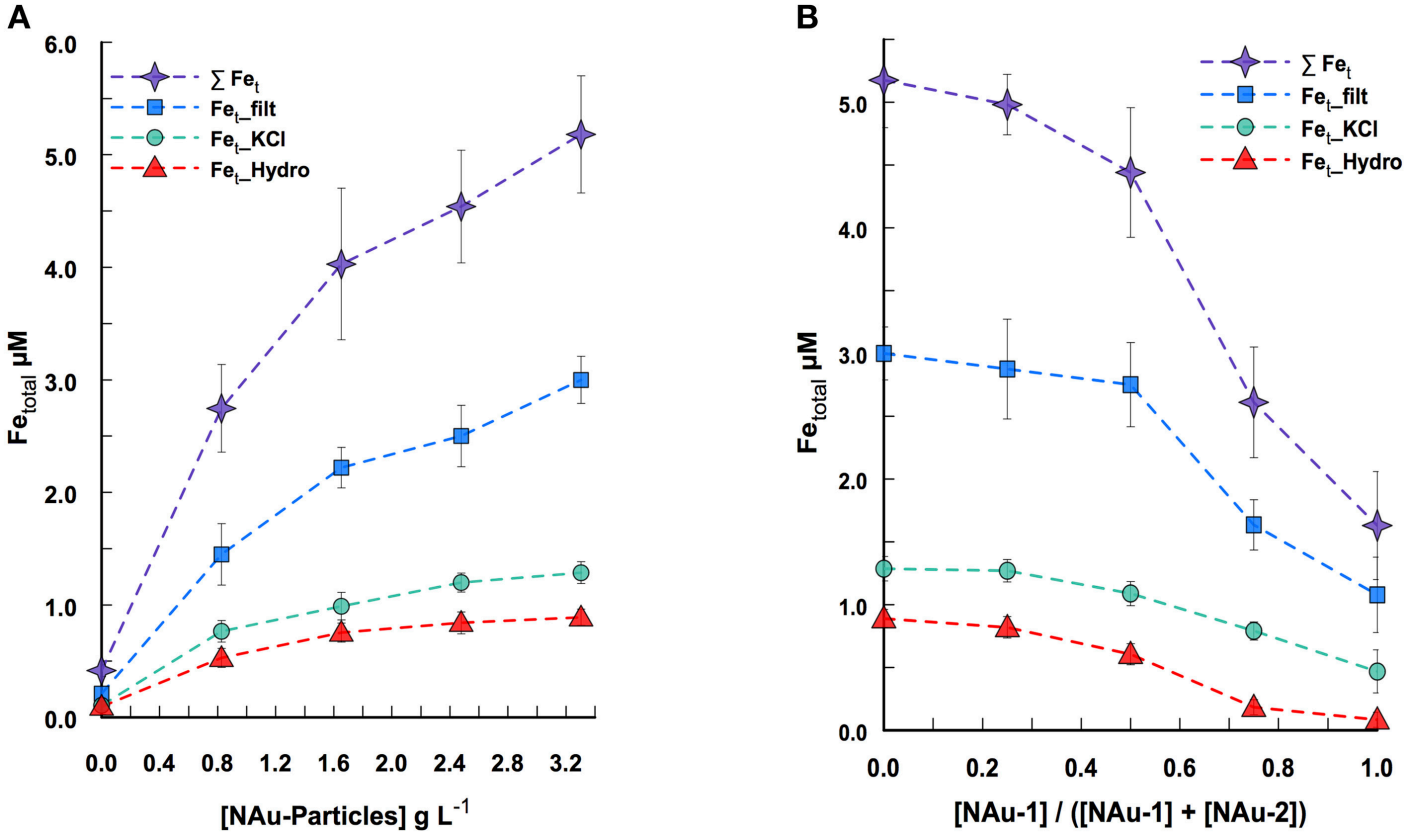
*****P. aeruginosa***-promoted Fe dissolution from silica gel-embedded nontronites in a system where NAu-1 to NAu-2 ratios are varied across a range of particle concentration, after 24 h of incubation. (A)** Fe dissolution for a constant NAu-1 to NAu-2 ratio ([NAu-1]/([NAu-1] + [NAu-2]) = 0) while the particle concentration varied. **(B)** Fe dissolution for a constant particle concentration (3.3 g L^−1^) with different NAu-1 to NAu-2 ratios. The symbols represent soluble Fe (squares), KCl extracted Fe (circles), hydroxylamine-KCl extracted Fe (triangles) and the sum of the three above Fe concentrations (stars). Error bars indicate the standard error of the mean (*n* = 2 for soluble Fe; *n* = 3 for KCl- and hydroxylamine-KCl extracted Fe).

**Figure 2:**
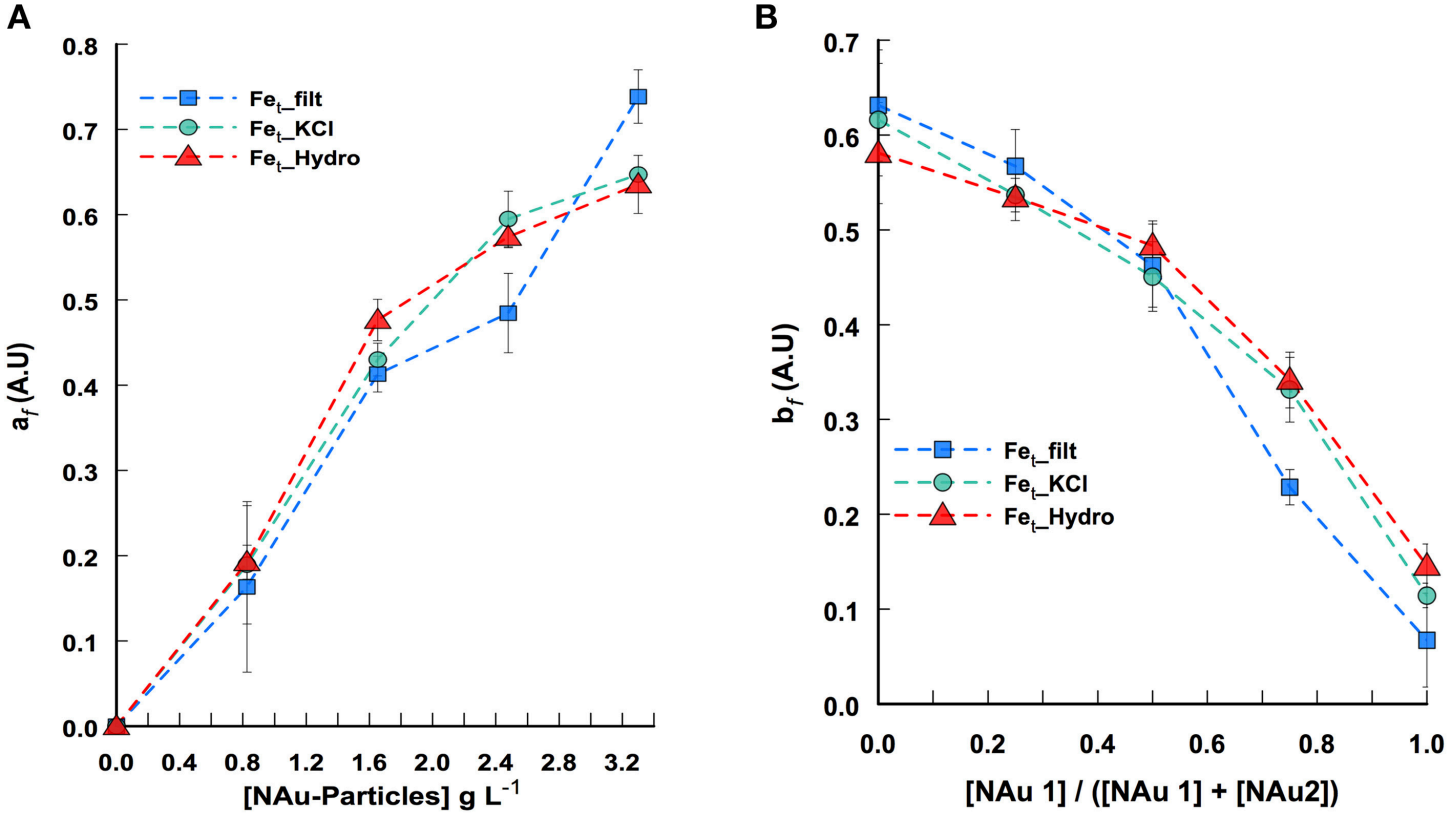
**Pattern of iron release determined from Candecomp/Parafac decomposition of the 3-way data set. (A)** Fe dissolution as a function of all ratio tested (1 ≥ [NAu-1]/([NAu-1] + [NAu-2] ≥ 0) across a range of particle concentration. **(B)** Fe dissolution as a function of all particle concentrations (0–3.3 g L-1) tested across a range of NAu-1 and NAu-2 ratios. a_*f*_ and b_*f*_ indicate the contribution of the ferrospectral-Fe complex signal to the global absorbance at any nontronite concentration and at any NAu-1 to NAu-2 ratio, respectively. The symbols represent the profile of soluble Fe (squares), KCl extracted Fe (circles), hydroxylamine-KCl extracted Fe (triangles). Error bars indicate the standard error of the mean (*n* = 2).

The multi-way approach consisted of 25 independent assays where the nontronite particle concentrations and NAu-1/NAu-2 ratio were varied all together and incubated with *P. aeruginosa* for 24 h before spectrophotometric measurement of iron release. For instance, Figure 1A represents the Fe dissolution profile when the assays were run with a constant nontronite ratio (100% of NAu-2 with [NAu-1]/[NAu-1] + [NAu-2] = 0%) across a range of particle concentrations (0 to 3.3 g L^−1^) in the presence of PAO1 WT. Figure 2A, on the other hand, illustrates the Fe dissolution profile when the assays were run with a constant particle concentration (i.e., 3.3 g L^−1^) while varying NAu-1 and NAu-2 ratio (i.e., 0 ≤ [NAu-1]/[NAu-1] + [NAu-2] ≤ 100%) in the presence of the same strain. An increase of Fe released in the culture medium was observed with increasing particle concentration indicating that the diffusion of the compounds responsible for the dissolution of Fe, through the porous silica gel was not a limiting factor in the range of tested particle concentrations (Figure 1A). For the assay run with the highest particle concentration (i.e., 3.3 g L^−1^) a total of 5.18 ± 0.21 μM of Fe were released in the culture media, 58% of which were soluble Fe. The same trend (soluble over total Fe) was noted across the entire particles concentrations tested (Figure 1A). The presence of Fe extracted with KCl and hydroxylamine treatment indicated that a small fraction of iron was adsorbed and/or precipitated on the mineral and/or on the silica gel after dissolution (Figure 1A). Overall the results show that a substantial amount of Fe was liberated in the culture media. Given the fact that NAu particles are highly insoluble at circumneutral pH, the Fe present in the media was likely chelated by organic molecules (i.e., excreted by bacteria), thus becoming available for bacterial uptake. Similarly, the related strain *Pseudomonas mendocina* ymp requires micromolar concentration of Fe for optimal growth (Dehner et al., 2010). Likewise, the amount of Fe dissolved from NAu-2 in the culture media is sufficient to support *P. aeruginosa* growth (not shown).

A significant amount of the released Fe (≈60%) was present in solution for the different ratios tested, indicating that the variation of NAu-1 and NAu-2 ratio did not increase or affect the adsorbed and/or the precipitated Fe fraction (Figure 1B). A two-trend pattern was observed during the release of Fe while varying the ratio: (i) in the ratio range of 0% ≤ [NAu-1]/[NAu-1] + [NAu-2] ≤ 50 %, the total amount of dissolved Fe decreased from 5.18 ± 0.21 μM to 4.44 ± 0.33 μM (i.e., a decrease of ≈ 15%) and (ii) in the range of 50 % ≤ [NAu-1]/[NAu-1] + [NAu-2] ≤ 100 % a higher decreased was registered from 4.44 ± 0.33 μM to 1.63 μM ± 0.30 (i.e., a decrease of ≈ 65%). The data indicate that *P. aeruginosa* did not mobilize as much Fe in the presence of NAu-1 than NAu-2. Because NAu-1 and NAu-2 were in a colloidal form in the silica gel and had almost the same surface area, their respective reactivity would not be affected by a change of surface area due to aggregation. Thus, the difference of reactivity observed between the two forms of nontronite is based on their crystallography distinctiveness.

To understand the general behavior of iron release by bacterial activity, it is necessary to consider all together the 25 independent assays run in this study by varying both NAu-1/NAu-2 ratio and the particle concentration in order to infer a biodissolution pattern. To this end, the absorbance spectra obtained from each assay were arranged in a 3-way data set (Nontronite concentration x NAu-1/NAu-2 ratio × absorbance spectra) and decomposed with the Candecomp/Parafac method (Figure S1). This approach allows singling the spectrum of interest out from the important absorbance background noise resulting from the different components present in the media and their possible interactions. Thus, the decomposition led to the extraction of the mixture coefficients a_*f*_ and b_*f*_ related to the contribution of the ferrospectral-Fe complex signal to the global absorbance signal at any nontronite concentration and at any [NAu-1]/[NAu-1] + [NAu2] ratio respectively. The iron release patterns from NAu-1 and NAu-2 during the incubation with *P. aeruginosa* PAO1 WT are presented in Figure 2. Overall, the data confirm that (i) iron is released from nontronites in a particle concentration-dependent manner (Figure 2A) and (ii) NAu-2 released substantially greater (approximately 3- to 4-fold) amounts of iron than NAu-1 (Figure 2B).

The same multi-way experiments were run with PAO1 Δ*pvdA*, a mutant strain unable to synthesize pyoverdine. By contrast to the WT strain, the dissolved Fe was below the detection limit in most of the 25 assays. The fact that no iron was detected after KCl and hydroxyalamine-KCl extraction suggested that the absence of detectable Fe in solution could not be explained by the adsorption or precipitation of Fe on the mineral and/or the silica gel surface. The data imply that the mutant strain was not capable to dissolve significant amounts of Fe(III) from NAu-1 or NAu-2 in our experimental set up (no physical contact between the bacteria cells and the mineral).

### Relative contribution of organic acids and siderophores to the iron dissolution process

#### Organic acids profiles

**Figure 3:**
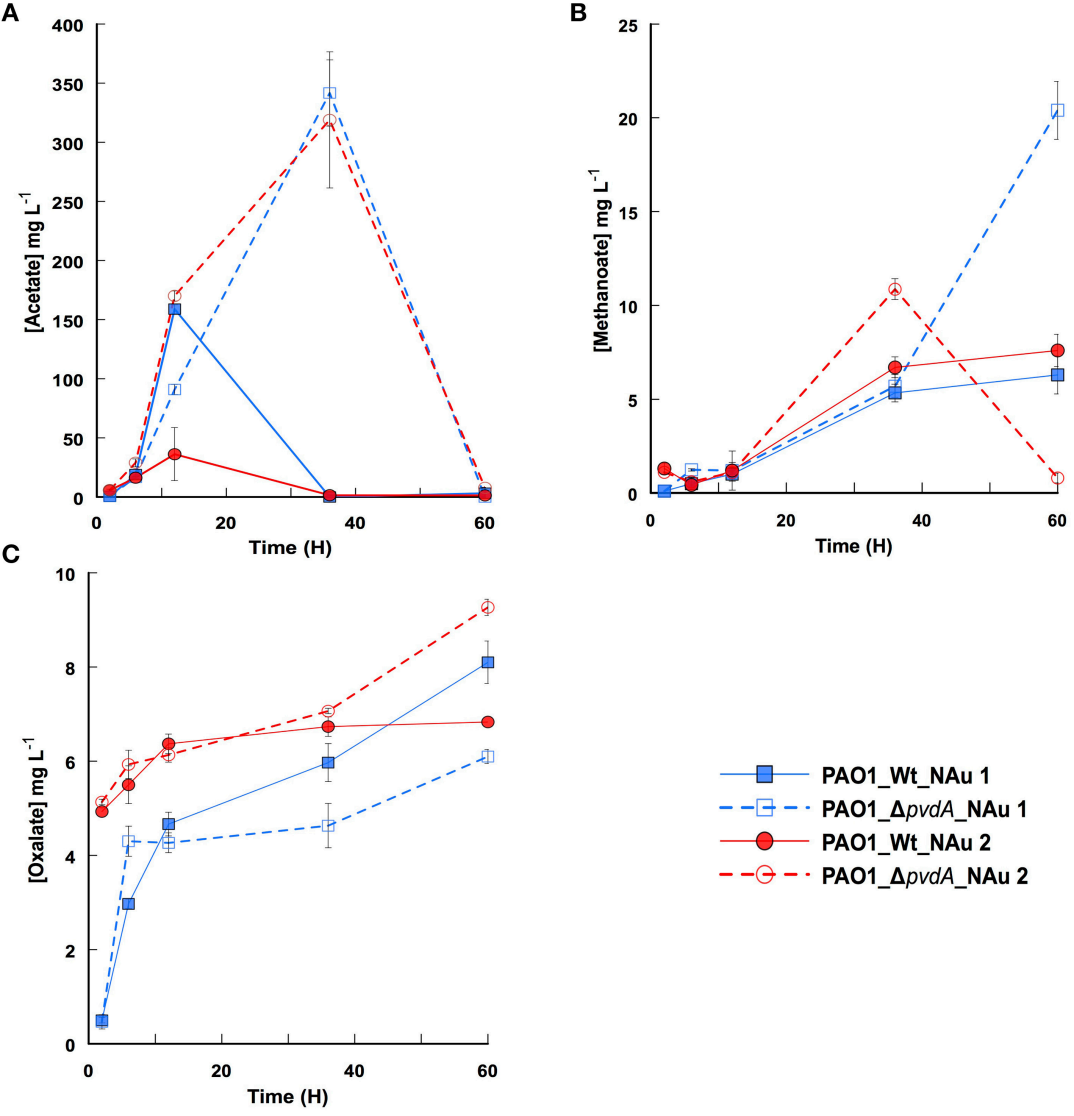
**Time profiles of acetate (A), methanoate (B), and oxalate (C) production by PAO1 WT (filled symbols) and PAO1 Δ***pvdA*** (empty symbols) in a medium containing NAu-1 (squares) and NAu-2 (circles)**. Values are means of triplicate measurements.

As shown previously, bacteria can close the concentration gap between mineral solubility and Fe requirement by solubilizing partially iron containing minerals. This process can be achieved by lowering the external pH and/or by the excretion of low molecular weight organic acids able chelate iron (Balland et al., 2010). The analysis of the culture media during the incubation time revealed the presence of oxalate, methanoate, and acetate (Figure 3). Acetate was the main organic molecule released, up to 340 mg L^−1^, while less than 10 mg L^−1^ of oxalate was present in the media. The results point out that PAO1 WT and PAO1 Δ*pvdA* produced the same low molecular weight organic molecules regardless the crystallographic structure of nontronite (NAu-1 vs. NAu-2). Despite some variations in organic acids profiles (e.g., higher level of acetate released by PAO1 Δ*pvdA* compared to the wild-type strain; Figure 3A), the solubilisation of iron cannot be attributed solely to the production of these organic compounds as no Fe was detected in the assays run with PAO1 Δ*pvdA*. However, we cannot rule out the possibility that they play a role in the dissolution process in the presence of siderophores. Indeed, it has been proposed that oxalate act in conjunction with siderophores and such synergic effect facilitates the dissolution of Fe from sparingly soluble minerals (Kraemer, 2004; Dehner et al., 2010).

#### Patterns of pyoverdine production

**Figure 4:**
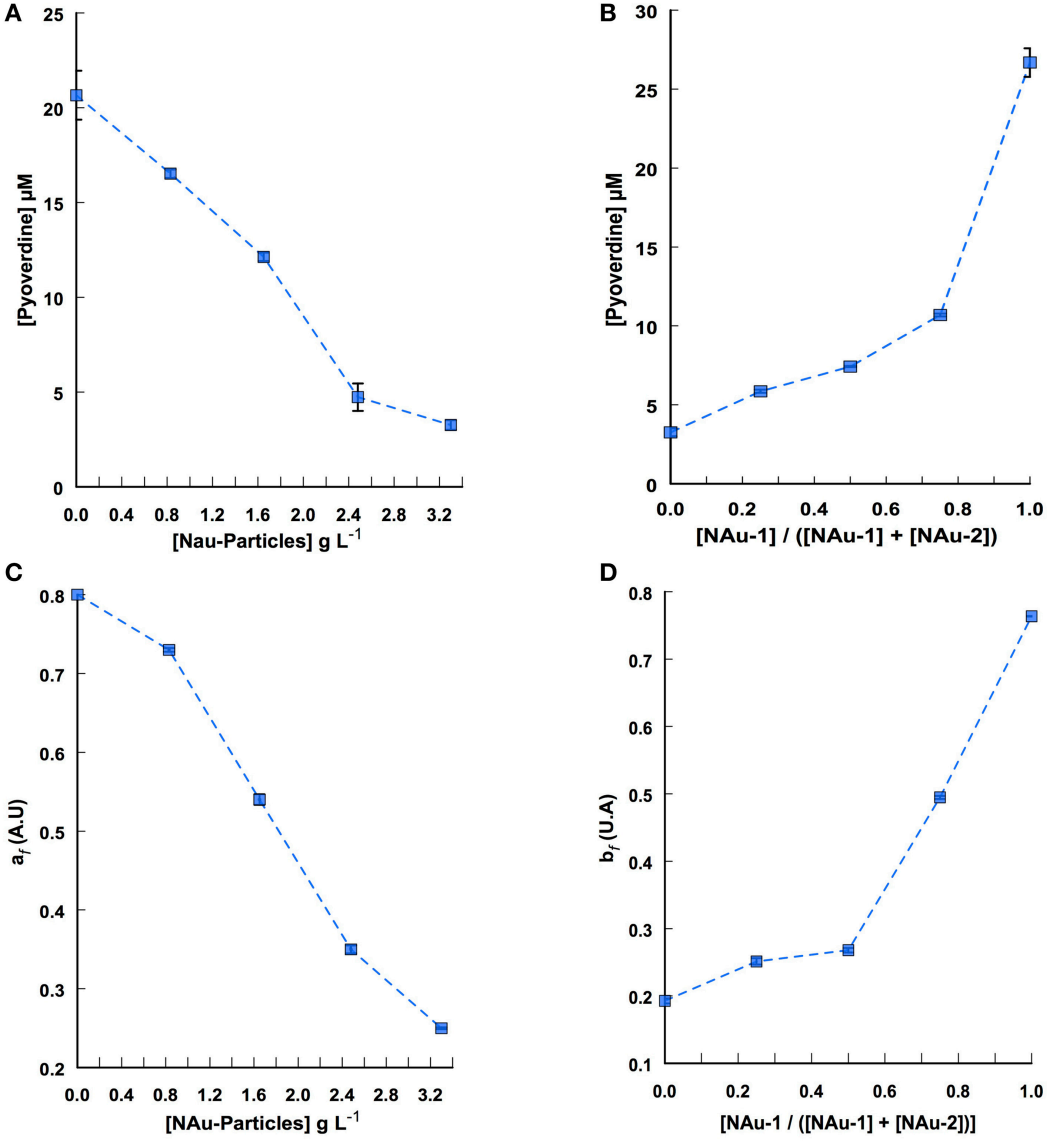
**Pyoverdine production by ***P. aeruginosa*** PAO1 incubated for 24 h in DCAA medium with increasing nontronite particle concentration and variables NAu-1 to NAu-2 ratios. (A)** Pyoverdine production in assays where the NAu-1 and NAu-2 ratio is kept constant ([NAu-1]/([NAu-1] + [NAu-2]) = 0) while the particle concentration is varied. **(B)** Pyoverdine production in assays where the particle concentration is kept constant (3.3 g L^−1^) with different NAu-1 to NAu-2 ratios. **(C)** Pattern of pyoverdine production as a function of all tested ratios over a range of particle concentrations, determined from CP decomposition of the 3-way data set. **(D)** Pattern of pyoverdine production as a function of all particle concentrations tested over a range of NAu-1 and NAu-2 ratios, determined from CP decomposition. a_*f*_ and b_*f*_ indicate the contribution of the pyoverdine signal to the global absorbance signal at any nontronite concentration and at any NAu-1 to NAu-2 ratio, respectively. Error bars represent the standard error of the mean (*n* = 2), most of them are within the size of the symbols.

An important route for Fe acquisition in bacteria is the excretion of siderophores. The production of pyoverdine by PAO1 WT was measured in the 25 independent assays. As an example, Figures 4A,B display pyoverdine production profile when the assays were run with a constant nontronite ratio (100% of NAu-2) across a range of particle concentration and with a constant particle concentration (i.e., 3.3 g L^−1^) while varying NAu-1 and NAu-2 ratio, respectively. The highest pyoverdine production level (20 ± 1.29 μM) was observed in the assays run without Fe (no added mineral) (Figure 4A). On the contrary, a decrease of about 88% was observed for the higher particle concentration showing that pyoverdine secretion is related to Fe deficient conditions. The assays run with different NAu-1/NAu-2 ratios indicated that the concentration of pyoverdine reached 26.26 ± 0.91 μM in the presence of 100% of NAu-1 (Figure 4B). In contrast, this concentration was 8-fold less when the Fe source was 100% of NAu-2. Pyoverdine secretion and Fe release (Figure 1) profiles were similar, thus supporting the critical role played by pyoverdine during the mobilization of structural Fe(III) as reported previously (Ferret et al., 2014). Additionally, the largest concentration of pyoverdine was measured when 100% of NAu-1 was used as the sole source of Fe. This result is in agreement with our previous observation (Figure 1) and fully supports our hypothesis that structural Fe(III) is more bioaccessible from NAu-2 than from NAu-1.

The absence of dissolved Fe in the culture medium when the experiments were run with *P. aeruginosa* PAO1 Δ*pvdA* and the fact that the same organic acids were produced by the mutant and the wild type cells clearly indicated that pyochelin and organic acid alone were not sufficient to dissolve Fe(III) from nontronite. Although Fe release was observed in the presence of pyoverdine (Figures 1, 4), our results did not allow to conclude whether the observed dissolution was only due to pyoverdine or to the synergic action of pyoverdine, pyochelin and organic acid.

Similar to our approach developed for the analysis of the global profile of Fe release, the absorbance spectra obtained from each assay were arranged in a 3-way data set (absorbance spectra × nontronite concentration × NAu-1/NAu-2 ratio) from which the pyoverdine secretion pattern can be described after spectral CP decomposition (Figure S1). The decomposition led to the extraction of the mixture coefficients a_*f*_ and b_*f*_ related to the contribution of the pyoverdine signal to the global absorbance signal at any nontronite concentration and at any [NAu-1]/[NAu-1] + [NAu2] ratio, respectively (Figures 4C,D). The pyoverdine production profile a_*f*_ as the function of particle concentration at any NAu-1/NAu-2 ratio decreased with particle concentration (Figure 4C). The pattern of pyoverdine secretion b_*f*_ for the different NAu-1/NAu-2 ratio tested at any particle concentration indicated a slight increase in the range of 100 to 50% of NAu-2 and a sharp rise, with the highest production in the presence of 100% of NAu-1 (Figure 4D), which is in well line with the iron dissolution profile determined above (Figure 2B). Even qualitative, the CP analysis provides a means of displaying the general profile of pyoverdine secretion in which the combined effect of both particle concentration and NAu-1/NAu-2 ratio variation were taken into account. Overall these results indicate that the mixing of NAu-1 and NAu-2 at different ratios did not affect the pyoverdine production profile due to a combined effect between the two minerals that could have been different from the sum of the individual effect.

### Abiotic dissolution of iron

**Figure 5:**
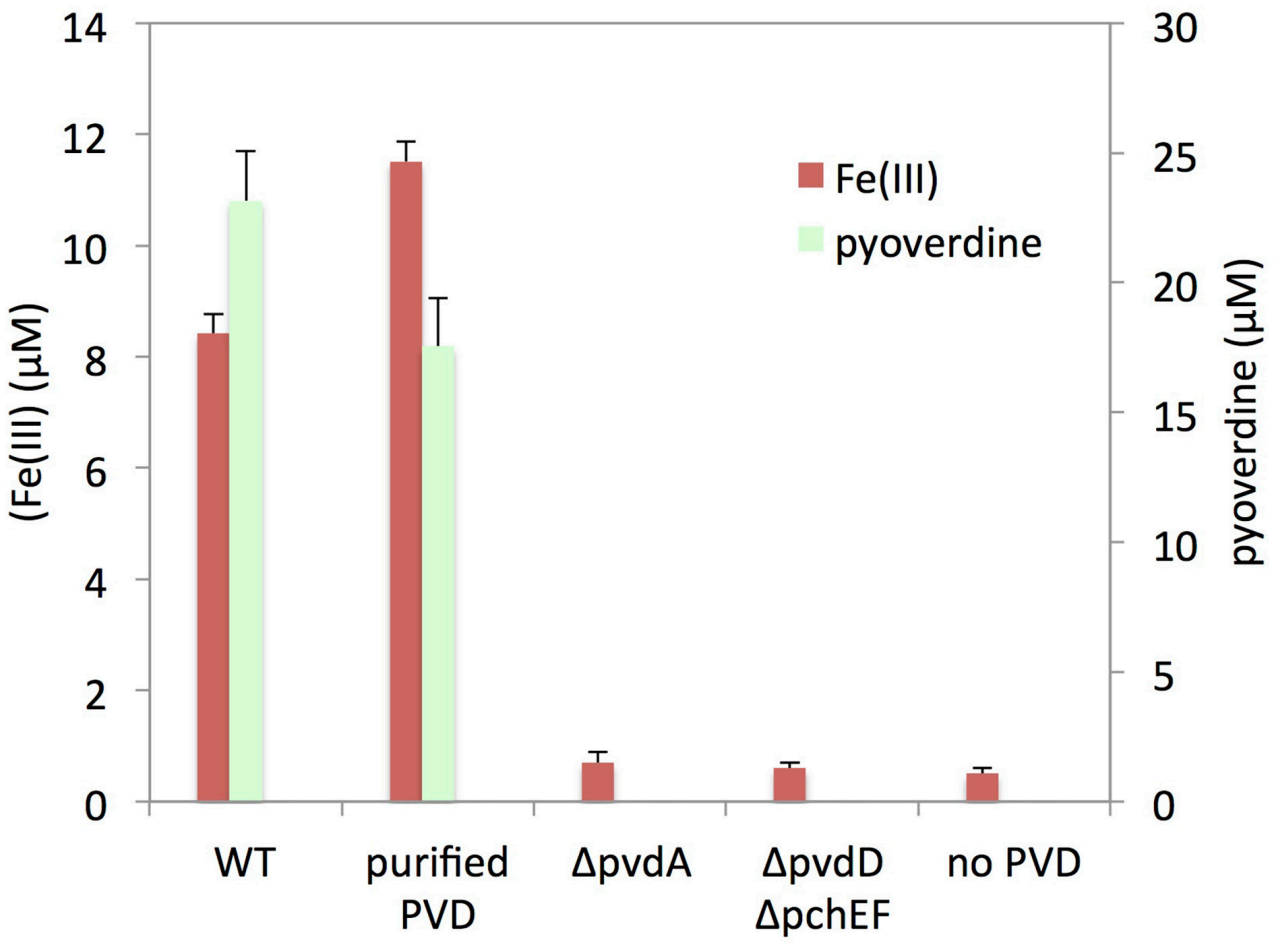
**Pyoverdine-mediated iron release from NAu-2**. Silica gel embedded NAu-2 particles were incubated with or without purified pyoverdine (PVD) and with cell-free culture supernatants of PAO1 WT, PAO1 Δ*pvdA* or PAO1 Δ*pvdD* Δ*pchEF* strains grown in DCAA medium. Pyoverdine and iron concentrations were determined after 24 h of incubation. Errors bars represent the standard error of the mean (*n* = 3).

To further investigate the role of pyoverdine in the mobilization of iron from nontronite, we performed batch experiments where silica gel plugs containing NAu-2 were incubated in iron deficient DCAA medium supplemented with purified pyoverdine, as well as in cell-free culture supernatants of PAO1 WT grown in DCAA. The analysis of the assay media after 24 h of incubation revealed that iron was mobilized in both cases (Figure 5). Comparable initial concentrations of pyoverdine in the assays led to the same level of iron dissolution, indicating that organic acids, pyochelin or other metabolite in the culture supernatant did not significantly contribute to mineral weathering. This was confirmed by the absence of detectable iron in assays performed with culture supernatants of the pyoverdin deficient mutant PAO1 Δ*pvdD* and the double pyochelin- and pyoverdine-deficient mutant (PAO1 Δ*pvdD* Δ*pchEF*). Overall, these data unambiguously show that pyoverdine is the main agent involved in weathering of nontronite in the absence of physical contact with bacterial cells.

A unique advantage of our bioweathering assay is that the size-defined, monolayer nontronite particles are homogeneously dispersed in the silica gel matrix, meaning they are evenly accessible to bacterial weathering metabolites. This feature makes it possible to infer a possible dissolution scenario from the knowledge of nontronite structure and above experimental data. Actually, in weathering assays run with NAu-2, only about 0.1% of the total structural iron was released in the medium, which corresponds to iron accessible from mineral edge sites. Previous acid dissolution experiments (e.g., Bickmore et al., 2001; Grybos et al., 2010) showed that the edge surfaces of phyllosilicates, characterized by groups with broken bonds, are much more reactive than the basal planes, which are built up with charge-satisfied and stable siloxane bonds. Edge surface sites also appear to be preferentially attacked by organic ligands (Bray et al., 2015; Zhang and Jun, 2015). This suggests that siderophore-promoted nontronite dissolution is dominated by cristal edges. In addition, the more extensive iron release from NAu-2 also is most likely due to a higher occurrence of tetrahedral Fe(III) in the structure compared to NAu-1 (8 and 2% respectively). We presume either that Fe(III) in tetrahedra is more accessible to chelating siderophores than in octahedra, or that dissolution of tetrahedral Fe(III) promotes iron release form adjacent octahedron sites via a yet unidentified mechanism.

Here we chose to work with a given particle size to assess the biological weathering processes independently of the possible contribution of basal to edge surface ratio, which depends on particle size (Grybos et al., 2011). The impact of different particle sizes on the extent of iron dissolution is now being investigated to define whether siderophores have a preferential reactive site.

### Effect of physical contact on Fe acquisition

**Figure 6:**
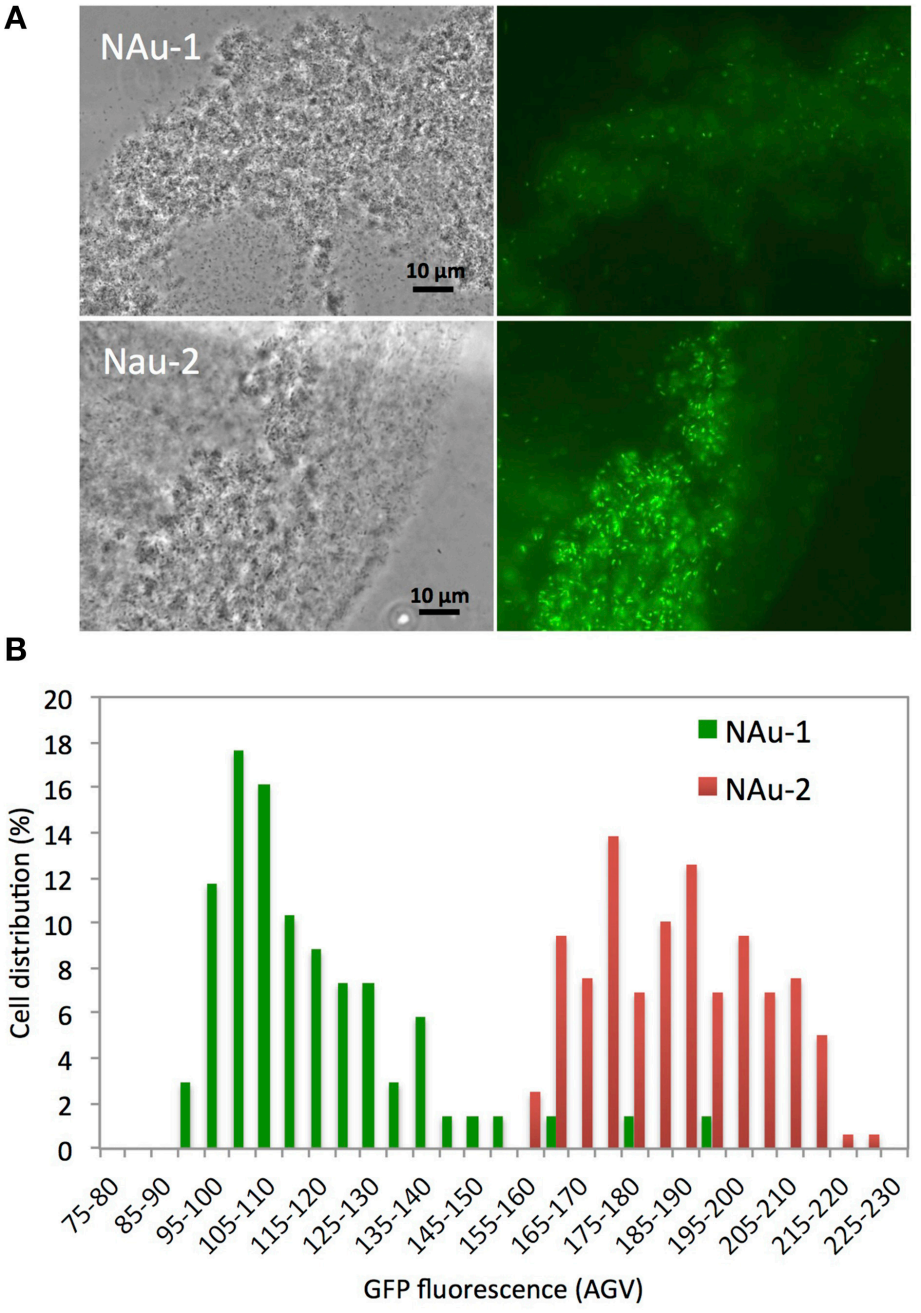
**Fluorescence of siderophore deficient ***P. aeruginosa*** strain PAO1 Δ***pvdD*** Δ***pchEF*** containing an iron regulated ***bfrB***-***gfp*** gene fusion incubated in direct contact with nontronite particles. (A)** Representative transmission (left panels) and fluorescence (right panels) micrographs showing aggregation of bacteria with NAu-1 (top panels) and NAu-2 (bottom panels) particles. **(B)** Expression of GFP in individual cells determined from analysis of images captured by fluorescence microscopy.

**Figure 7:**
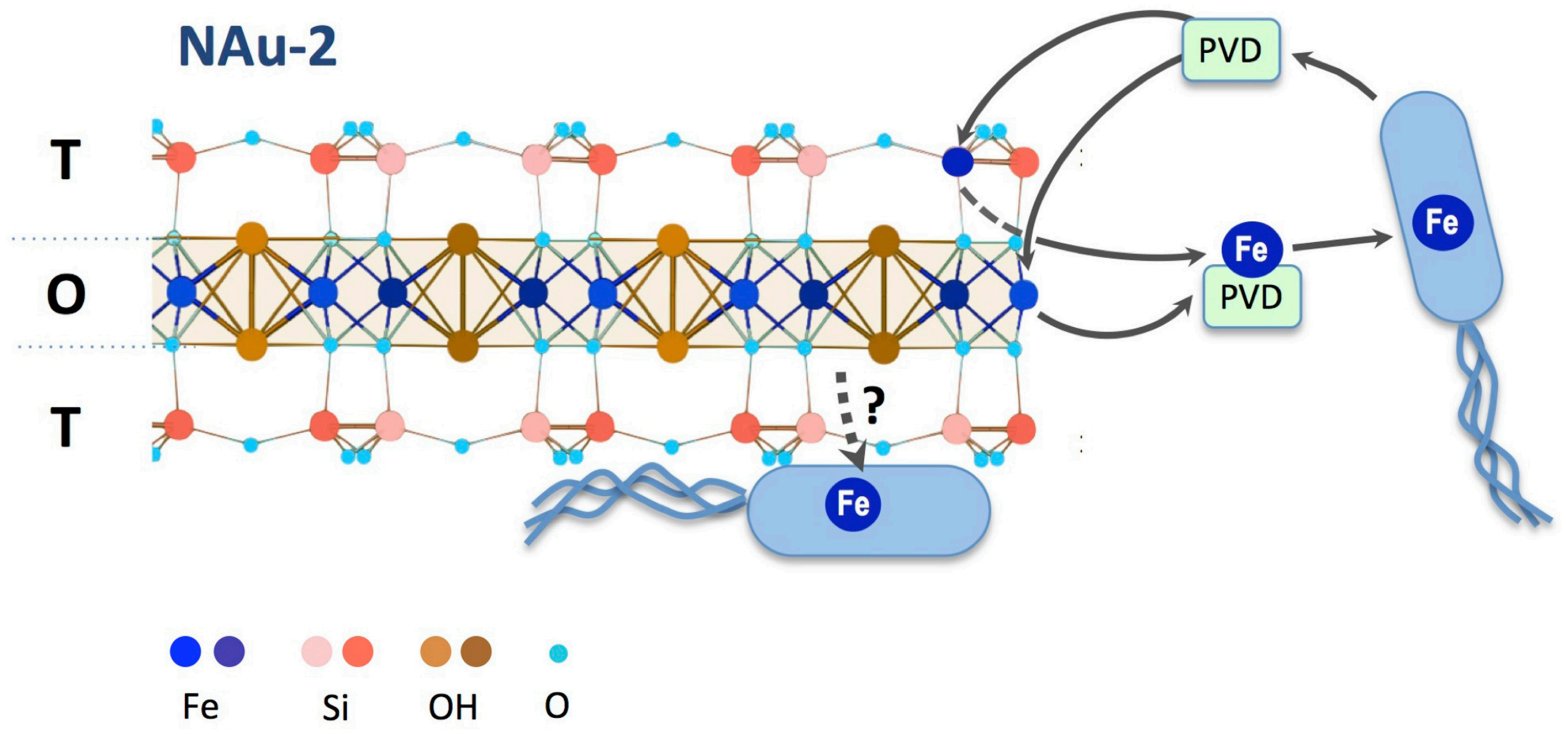
**Proposed mechanism of iron mobilization from nontronite**. Under iron deficient conditions, *P. aeruginosa* produces pyoverdine that chelates and solubilizes Fe(III) from edge sites of octahedral (O) or tetrahedral (T) sheets. The higher occurrence of tetrahedral Fe(III) sites in NAu-2 facilitates iron mobilization compared to NAu-1. Iron from pyoverdine-Fe^3+^ complexes can be taken up into the bacterial cell to sustain growth. When in direct contact with the mineral particles, *P. aeruginosa* can acquire iron via a siderophore-independent mechanism.

To test whether a direct contact would alleviate the need for pyoverdine, a *bfrB*-*gfp* gene fusion constructed in pPROBE-NT was introduced into *P. aeruginosa* PAO1 Δ*pvdD* Δ*pchEF*, The expression of the *bfrB* gene encoding a bacterioferritin involved in iron storage is induced under iron-replete conditions and, therefore, high expression levels of this gene would indicate that the cells satisfied their metabolic Fe requirement (Parrello et al., 2015). *P. aeruginosa* Δ*pvdD* Δ*pchEF*/pPROBE-bfrB was incubated in direct contact with NAu-1 and NAu-2. The induction of *bfrB* gene expression was estimated by measuring the GFP fluorescence after 24 h of incubation (Figure 6). The images of bacterial cell aggregates show an expression of *bfrB* gene in the presence of nontronite, with a higher expression level when the experiment was run with NAu-2 (Figure 6B) compared to NAu-1. This reinforced our previous observations (Figures 1, 4) that the bioavailability of Fe is controlled by the crystallography of the mineral. Furthermore, the siderophore-deficient mutant was able to acquire Fe, which was not readily available for biological uptake, when a physical contact was established with the mineral. A simplified scheme for Fe release from iron bearing clays such as nontronites is proposed in Figure 7.

Previous work with a siderophore-deficient mutant of *Pseudomonas mendocina* ymp resulted in similar observations. The mutant strain was unable to acquire Fe from iron containing minerals without a physical contact (Dehner et al., 2011; Kuhn et al., 2013). However, the incubation of cells in the vicinity of the mineral led to the acquisition of Fe from the mineral phase, indicating that another metabolic strategy for iron uptake could be activated to alleviate the absence of siderophore. Indeed, Dehner and co-workers have demonstrated that *Pseudomonas mendocina* was able to reduce and withdraw Fe from montmorillonite, an iron containing clay mineral *via* a cell-associated enzyme. Similarly, *P. aeruginosa* could use a reduction-promoted Fe acquisition when the cells were in direct contact with Fe bearing minerals.

## Conclusion

*P. aeruginosa* is capable to acquire structural Fe(III) of a clay mineral, nontronite, through the use of siderophores when there is a physical barrier between the cells and the mineral particles. Furthermore, the mixing of NAu-1 and NAu-2 didn't affect neither the release of Fe(III) nor the production of siderophore indicating the absence of interaction between NAu-1 and NAu-2 as shown by our multi-parametric experimental approach coupled to CP spectral decomposition. Although *P. aeruginosa* can produce two siderophores, pyoverdine and pyochelin, pyoverdine is the key driver of Fe solubilisation. A *P. aeruginosa* mutant deficient in the production of both siderophores appears to acquire Fe from nontronite if a physical contact between the bacterial cells and the mineral is established. This second mechanism of Fe acquisition is still under investigation. Structural Fe(III) of NAu-2 was shown to be more bioaccessible than the one of NAu-1 independently of the Fe acquisition pathway (i.e., pyoverdine or direct contact-promoted Fe acquisition). Thus, the bioavailability of structural Fe(III) is governed by the crystallochemistry of the mineral. This observation leads to questions how the crystal chemical environment of Fe(III) (tetrahedron, octahedron) constrains the release of Fe and how siderophores interact with these sites. Furthermore, it is established that bacteria can influence layer charge, cation exchange capacity, swelling and the rheological properties of clay minerals through the production of siderophores. Therefore, additional work is needed with different particle sizes and different environmental clay samples to constrain how the change in their properties and behavior induced by bacterial activity impact the biogeochemical cycle of iron and associated trace elements.

## Author contributions

Conceived and designed experiments: DP, CM, PB, AZ. Performed the experiments: DP, CM, PB, AZ. Analyzed the data: DP, CM, PB, AZ. Wrote the paper: AZ, PB. All authors read and approved the final manuscript.

### Conflict of interest statement

The authors declare that the research was conducted in the absence of any commercial or financial relationships that could be construed as a potential conflict of interest.
