# Supplementary figures and images for: Siderophore-Mediated Iron Dissolution from Nontronites Is Controlled by Mineral Cristallochemistry
Source: Front Microbiol. 2016 Mar 31;7:423. doi: 10.3389/fmicb.2016.00423 (PMC4814481; doi:10.3389/fmicb.2016.00423)

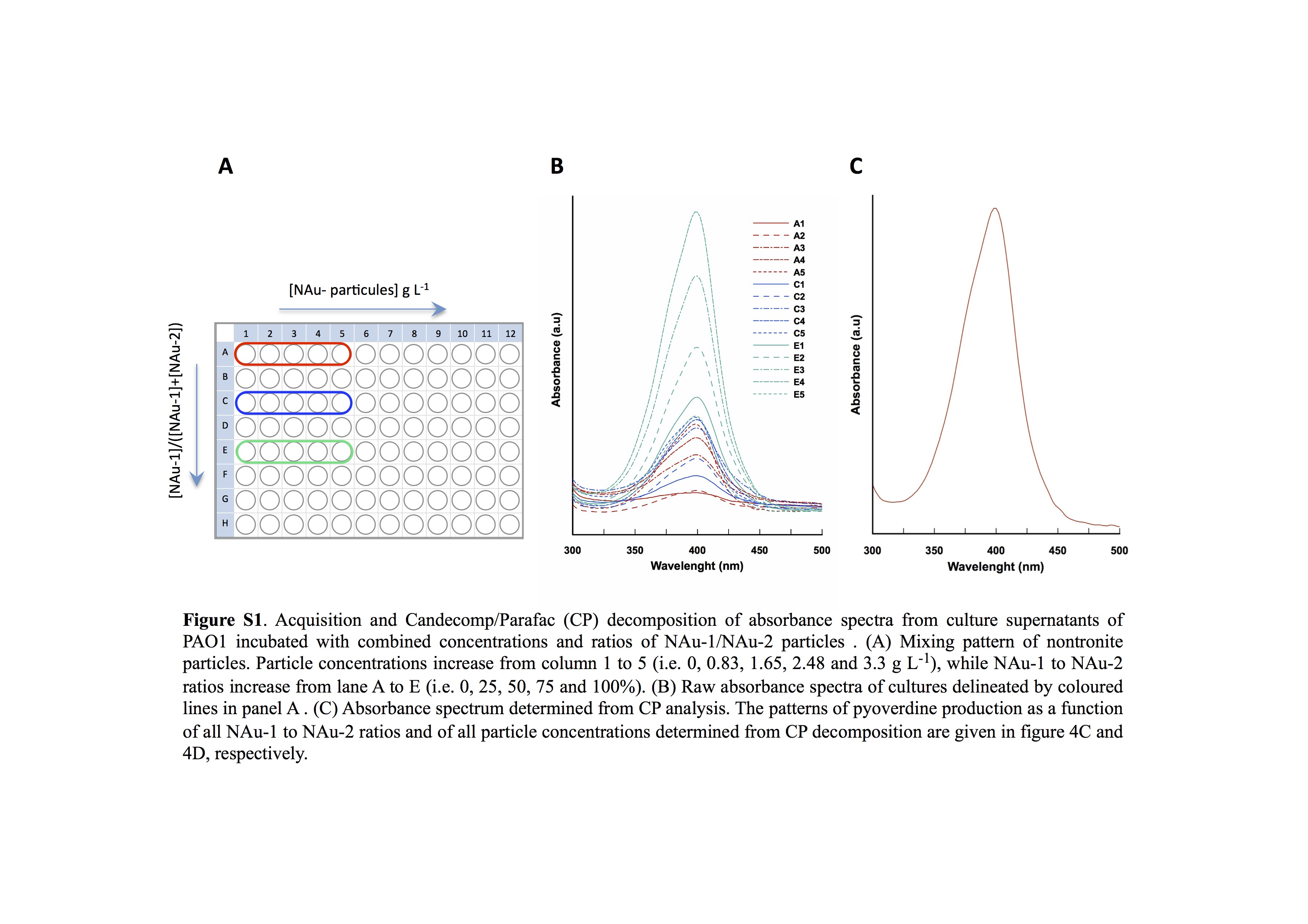

Supplement: Supplementary file 1 [file Image1.JPEG]
